# **Hypolipidemic, Hepatoprotective and Renoprotective Effects of** ***Cydonia Oblonga*** **Mill. Fruit in Streptozotocin-Induced Diabetic Rats**

**Published:** 2015

**Authors:** Mansur Mirmohammadlu, Seyed Hojjat Hosseini, Mohammad Kamalinejad, Majid Esmaeili Gavgani, Maryam Noubarani, Mohammad Reza Eskandari

**Affiliations:** a *Department of Pharmacology and Toxicology, School of Pharmacy, Zanjan University of Medical Sciences, Zanjan, Iran. *; b *Department of basic science, Science and Research branch, Islamic Azad University, Tehran, Iran. *; c *Faculty of Pharmacy, Shahid Beheshti University of Medical Sciences, Tehran, Iran.*; d *Department of Immunology, Faculty of Medicine, Tabriz University of Medical Sciences, Tabriz, Iran.*

**Keywords:** *Cydonia oblonga Mill*, Diabetes, Hepatoprotective, Hypolipidemic, Renoprotective

## Abstract

Diabetes mellitus is associated with complications in several different systems of the body, and the incidence of diabetes is rapidly increasing worldwide. The objective of the present study was to evaluate the effect of aqueous extract of *Cydonia oblonga* Mill. Fruit on lipid profile and some biochemical parameters in streptozotocin-induced diabetic rats. The extract showed anti hyper lipidemic activity as evidenced by significant decreases in serum triglyceride, total cholesterol, and low density lipoprotein cholesterol (LDL-C) levels along with the elevation of high density lipoprotein cholesterol (HDL-C) in the diabetic rats. The biochemical liver functional tests were also analyzed and it was shown that serum biomarkers of liver dysfunction, including alanine transaminase (ALT), aspartate transaminase (AST), and alkaline phosphatase (ALP) were significantly reduced in aqueous extract of *Cydonia oblonga* Mill. treated diabetic rats. In addition, our results showed that the oral administration of the extract prevented diabetes-induced increase in serum urea and creatinine levels as the markers of renal dysfunction. In conclusion, the present study indicates that aqueous extract of *Cydonia oblonga* Mill. Is able to improve some of the symptoms associated with diabetes and possesses hypolipidemic, hepatoprotective, and renoprotective effects in streptozotocin-induced diabetic rats.

## Introduction

Diabetes mellitus is a worldwide epidemic with considerable health and economic consequences that affects nearly 10% of the population (1, 2). This serious metabolic disorder remains a leading cause of cardiovascular diseases, blindness, end-stage renal failure, and hospitalizations. The disease characterized by chronic hyperglycemia and disturbances in fat and protein metabolism (3). Hyperglycemia is typically accompanied by progressive development of microvascular and macrovascular complications, causing morbidity and mortality in diabetic patients (4).

Diabetes is also associated with abnormalities in serum lipids and it was shown that elevated levels of total cholesterol, triglyceride, low density lipoprotein cholesterol (LDL-C) as well as low concentration of high density lipoprotein cholesterol (HDL-C) in diabetes are accompanied with coronary heart disease (5, 6). Therefore, lipid disorders should be quickly diagnosed and treated as a part of diabetes comprehensive treatment. Previous studies have also revealed that the liver is one of the main organs affected by diabetes and that this progressive disease may increase the risk of both chronic liver diseases and hepatocellular carcinoma (7-11). As a consequence, the activities of liver damage markers including, serum alanine aminotransferase (ALT), aspartate transaminase (AST), and alkaline phosphatase (ALP) are increased in the untreated diabetic patients (12). Diabetic nephropathy and renal dysfunction is one the diabetes related complications and approximately 20-40% of patients with diabetes (type 1 and type 2) develop nephropathy (13). As a result, the plasma levels of urea and creatinine are elevated in the diabetic hyperglycemia that is considered as significant markers of renal impairment (14).

Medicinal plants have become increasingly attractive agents in the treatment of diabetes and its related complications (15-17). *Cydonia oblonga* Mill.,commonly called quince is one of these plants that belongs to the Rosaceae family and is cultivated for fruit production all over the world. It is believed that quince originated in Northern Iran, Turkmenistan, and the far west regions of Anatolia and Greece (18). *Cydonia oblonga* Mill. Fruit is recognized as an important dietary source and contains polyphenolic compounds, organic acids, and free amino acids (19-21). *Cydonia oblonga* Mill. has been utilized to decrease symptoms of diabetes in Iranian and Turkish folk medicines and it was shown that the leaves of this plant has antioxidant, anti-hyperglycemic, hypolipidaemic, and hepatoprotective effects (22-23). Since hyperglycemia is accompanied by dyslipidemia and abnormalities in liver and kidney functions, the present study was designed to investigate the hypolipidemic, hepatoprotective, and renoprotective properties of *Cydonia oblonga* Mill. Fruit aqueous extract in streptozotocin-induced diabetic rats.

## Experimental

*Materials and Methods*

Plant material and preparation of the extract: *Cydonia oblonga* Mill. Fruits were collected from Shahriar, Alborz province of Iran. The collected fruits were scientifically approved by the Department of Botany, Shahid Beheshti University (Voucher number: 8054, deposited in: Shahid Beheshti University Herbarium). Fresh fruits were cleaned and then dried in the shade at room temperature. Fruits were decocted in water for 30 min. Then, the extract was filtered and concentrated to the desired level (honey-like viscosity), and stored at -20°C. The moisture level of the extract was determined as follows: 2 g of final extract was placed in an oven at 60–65°C for 72 h and then weighed. Weight loss was used as a moisture indicator. The final extract contained 24% water. This extract was dissolved in distilled water at the desired concentrations just before use (24). We chose a wide concentration range for aqueous extract of *Cydonia oblonga* Mill. Fruit in our pilot study and their inhibitory effects were evaluated (data not shown). By omitting non effective, poor effective or toxic concentrations, the 80, 160 and 240 mg/kg body weight were selected.

Chemicals: Streptozotocin was purchased from Sigma-Aldrich Co. (Taufkrichen, Germany). All other chemicals were of the highest commercial grade available.

Animals: Male Sprague-Dawley rats weighing 250 to 300 g were housed in ventilated plastic cages over PWI 8-16 hardwood bedding. There were 12 air changes per h, 12 h light photoperiod (lights on at 0800 h), an environmental temperature of 21–23°C and a relative humidity of 50–60%. The animals were fed a normal standard chow diet and given tap water *ad libitum*. Principles of laboratory animal care" (NIH publication No. 85-23, revised 1985) were followed. All experiments were conducted according to the ethical standards and protocols approved by the Committee of Animal Experimentation of Zanjan University of Medical Sciences, Zanjan, Iran.

Experimental induction of diabetes in rats: Experimental diabetes was induced following an overnight fast, by a single intraperitoneal (i.p.) injection of 60 mg/kg streptozotocin freshly dissolved in 1.0 ml citrate buffer (0.1 M, pH = 4.5) (25). Control animals were injected with vehicle only. Hyperglycemia was confirmed 4 days after injection by measuring the tail vein blood glucose level with a standardized glucometer (ARKAY, INC., Japan). Only the animals with fasting blood glucose levels ≥ 250 mg/dl were selected for the study.

Collection of blood samples: Blood samples were collected from the tail vein. Blood was collected and serum was immediately frozen and stored at -70°C until the biochemical determinations were performed.

Biochemical parameters: The serum levels of triglyceride, total cholesterol, HDL-C, ALT, AST, ALP, urea, and creatinine were determined by commercially available enzyme kits (Pars Azmoon, Tehran, Iran) and using an automatic analyzer (Architect c8000 Clinical Chemistry System, USA) (26). LDL-C was calculated according to Friedewald equation as follows: LDL-C = Total cholesterol-HDL-C- (Triglyceride/5) (27).

Protocol design: Normal and diabetic rats were divided into five groups of nine rats each (n = 9). Group I served as negative control (non diabetic); Group II served as positive control (diabetic but not treated); Group III, IV and V diabetic rats were treated with aqueous extract of *Cydonia oblonga* Mill. Fruit at 80, 160, and 240 mg/kg body weight respectively. Each plant extract sample was administered orally once daily using an intragastric tube for 6 weeks.

Statistical Analysis: Levene’s test was used to check the homogeneity of variances. The data were analysed using one-way analysis of variance (ANOVA) followed by Tukey’s HSD as the post hoc test. The results were presented as the mean ± SD (n = 9). The minimal level of significance chosen was *p* < 0.05.

## Results and Discussion

*Serum lipid profiles*

**Figure 1:**
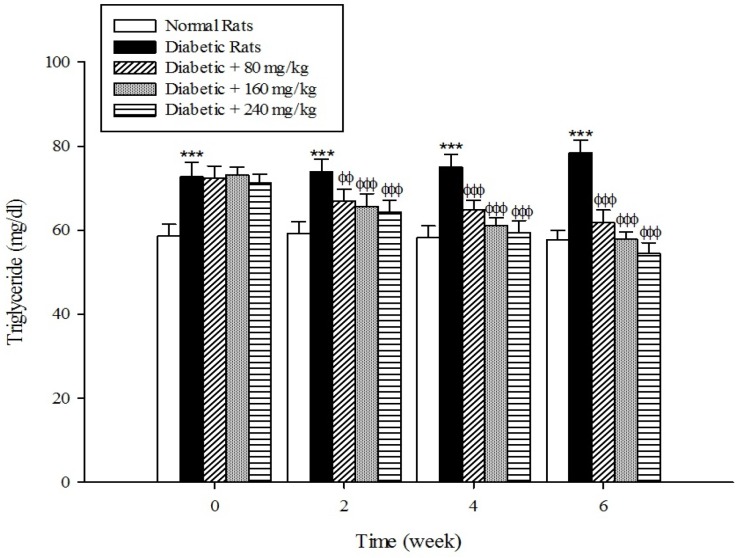
Effects of aqueous extract of *Cydonia oblonga* Mill. on serum triglyceride in streptozotocin-induced diabetic rats. Values are presented as mean ± SD (n = 9).

**Figure 2:**
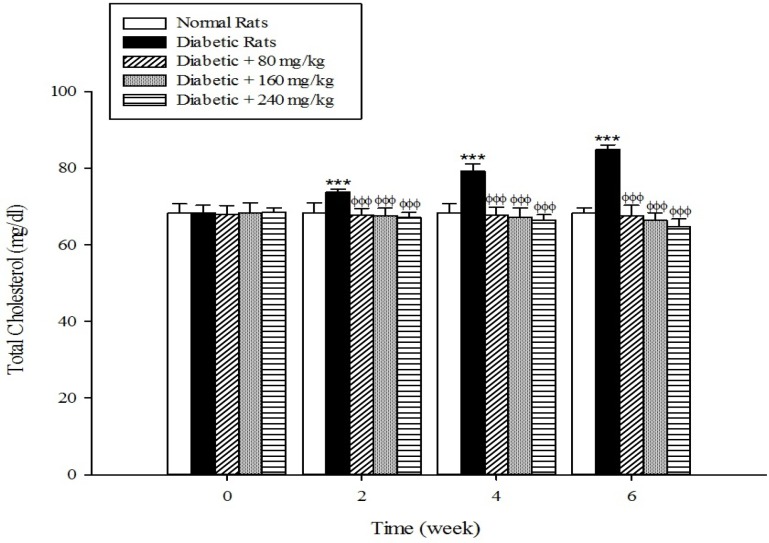
Effects of aqueous extract of *Cydonia oblonga* Mill. on total cholesterol in streptozotocin-induced diabetic rats. Values are presented as mean ± SD (n = 9).

**Figure 3:**
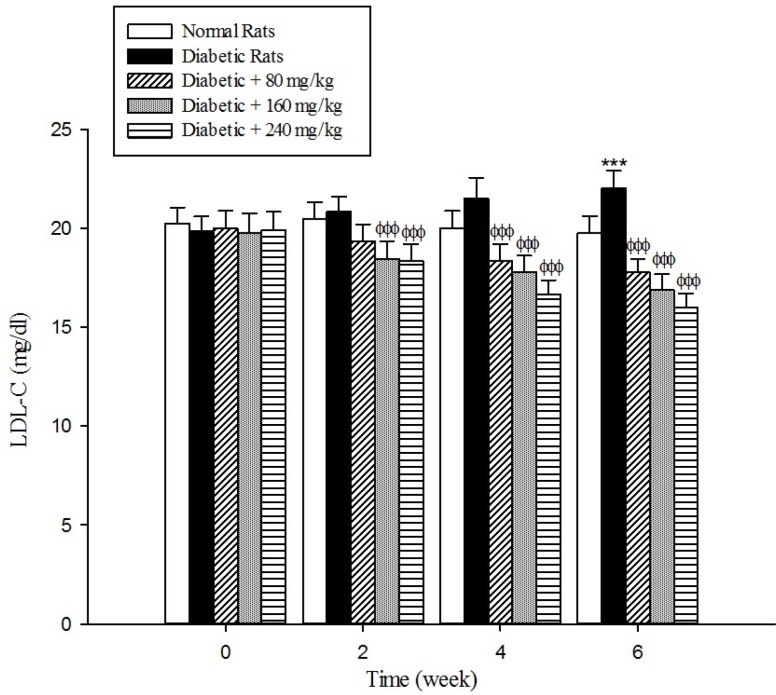
Effects of aqueous extract of *Cydonia oblonga* Mill. on LDL-C in streptozotocin-induced diabetic rats. Values are presented as mean ± SD (n = 9).

**Figure 4:**
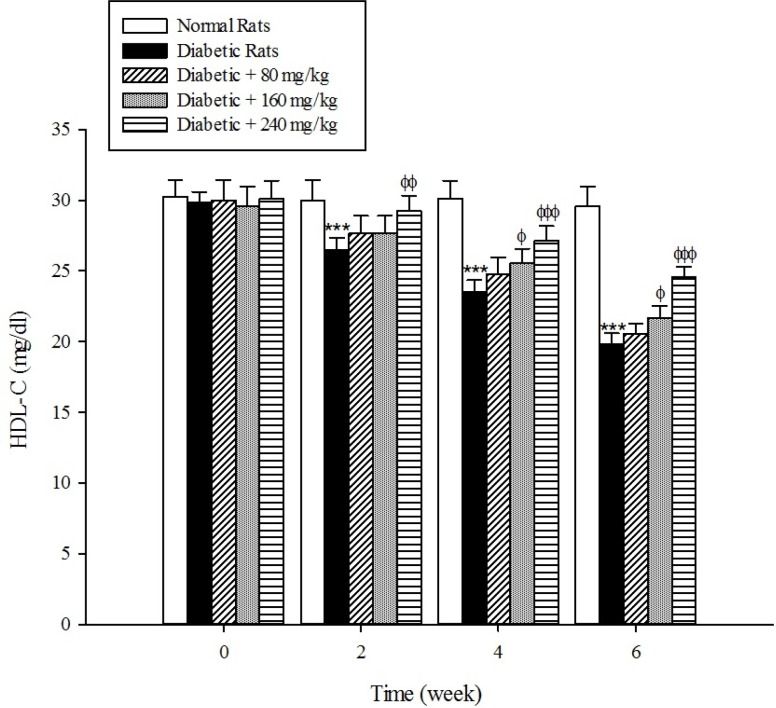
Effects of aqueous extract of *Cydonia oblonga* Mill. on HDL-C in streptozotocin-induced diabetic rats. Values are presented as mean ± SD (n = 9).

The results of the serum lipid profile showed that streptozotocin injection led to the development of hyperlipidemia in which serum triglyceride, total cholesterol, and LDL-C markedly (*p* < 0.001) increased when compared to the control group (Figure 1-3). However, HDL-C decreased in diabetic rats in comparison with the normal control (Figure 4). As shown in Figure1-4 the different concentrations of aqueous extract of *Cydonia oblonga* Mill. Fruit caused a significant decrease in the serum triglyceride, total cholesterol, and LDL-C, but a significant increase in HDL-C levels in the diabetic rats during 6 weeks of the treatment.

***p < 0.001 compared with normal rats and θθ *p *< 0.01; θθθ *p *< 0.001 compared with diabetic rats.

***p < 0.001 compared with normal rats and θθθ *p *< 0.001 compared with diabetic rats.

***p < 0.001 compared with normal rats and θθθ *p *< 0.001 compared with diabetic rats.

***p < 0.001 compared with normal rats and θ *p *< 0.05; θθ *p *< 0.01; θθθ *p *< 0.001 compared with diabetic rats.

*Liver parameters*

**Figure 5:**
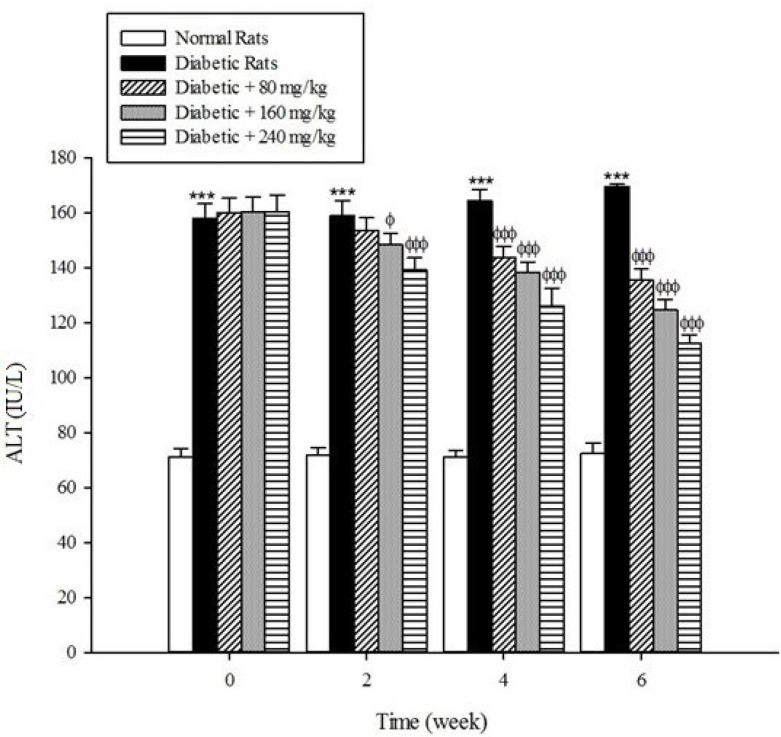
Effects of aqueous extract of *Cydonia oblonga* Mill. on ALT in streptozotocin-induced diabetic rats. Values are presented as mean ± SD (n = 9).

**Figure 6:**
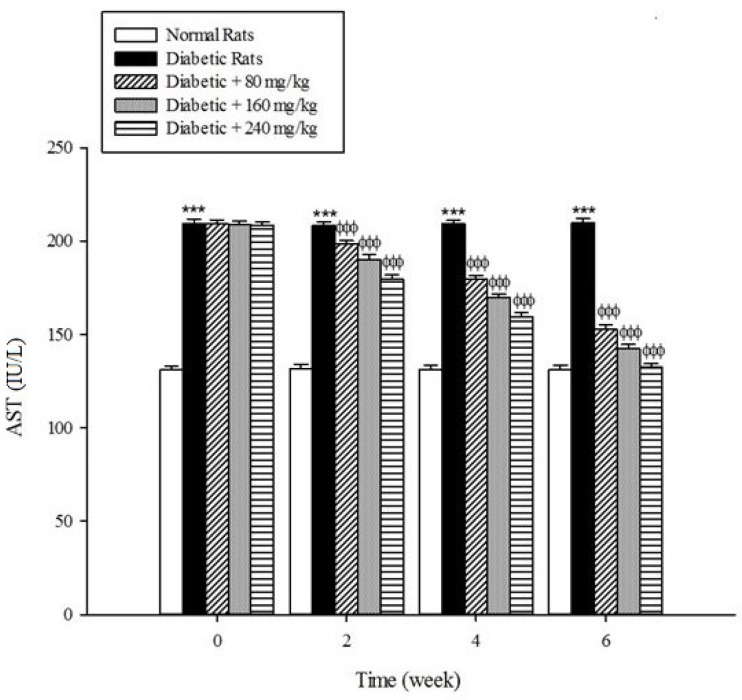
Effects of aqueous extract of *Cydonia oblonga* Mill. on AST in streptozotocin-induced diabetic rats. Values are presented as mean ± SD (n = 9).

**Figure 7:**
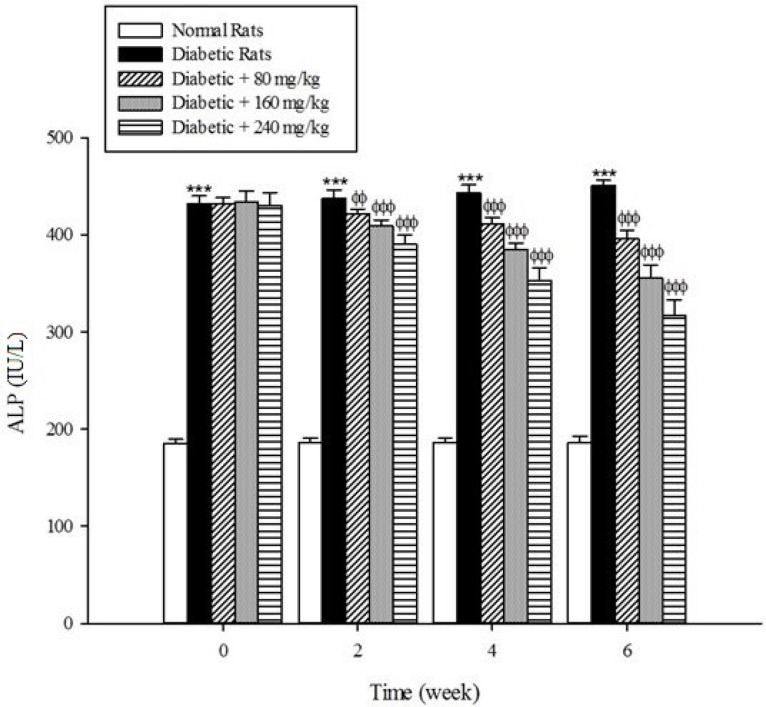
Effects of aqueous extract of *Cydonia oblonga* Mill. on ALP in streptozotocin-induced diabetic rats. Values are presented as mean ± SD (n = 9).

Serum activities of ALT, AST, and ALP as the markers of liver function significantly (*p* < 0.001) were increased in the untreated diabetic rats in comparison to the non-diabetic rats (Figure 5-7). The extract at the concentrations of 80, 160, and 240mg/kg caused a significant decrease in the biomarkers of liver injury in the diabetic rats treated with the extract (*p* < 0.001).

***p < 0.001 compared with normal rats and θ *p *< 0.05; θθθ *p *< 0.001 compared with diabetic rats.

***p < 0.001 compared with normal rats and θθθ *p *< 0.001 compared with diabetic rats.

***p < 0.001 compared with normal rats and θθ *p *< 0.01; θθθ *p *< 0.001 compared with diabetic rats.

*Kidney parameters*

**Figure 8:**
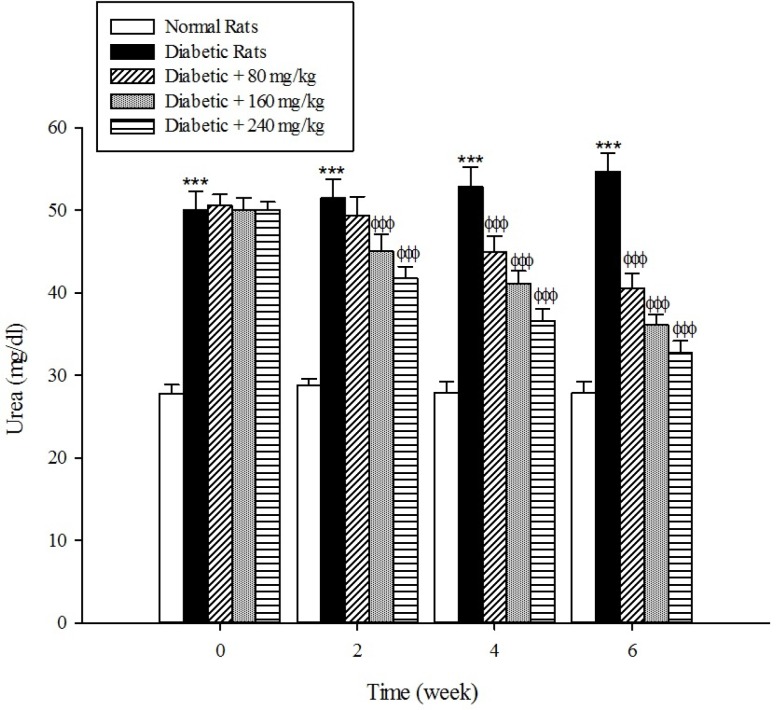
Effects of aqueous extract of *Cydonia oblonga* Mill. on urea in streptozotocin-induced diabetic rats. Values are presented as mean ± SD (n = 9).

**Figure 9:**
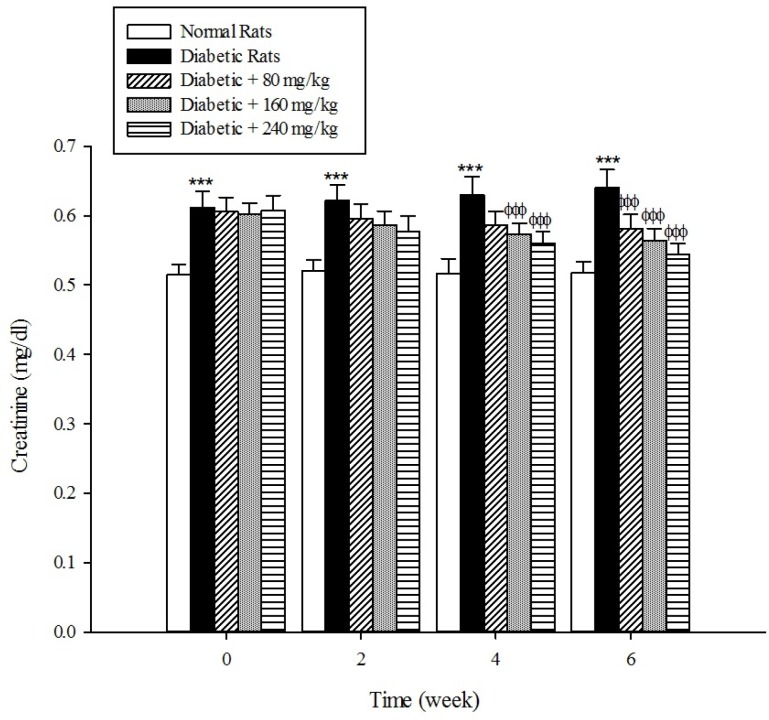
Effects of aqueous extract of *Cydonia oblonga* Mill. on creatinine in streptozotocin-induced diabetic rats. Values are presented as mean ± SD (n = 9).

The levels of serum blood urea and creatinine as the markers of renal dysfunction were elevated (*p *< 0.001) in the diabetic rats when compared with the normal rats (Figure 8, 9). As shown in Fig 8 and 9 the treatment of diabetic animals with the extract significantly inhibited an increase in the serum urea and creatinine concentrations (*p* < 0.001).

***p < 0.001 compared with normal rats and θθθ *p *< 0.001 compared with diabetic rats.

***p < 0.001 compared with normal rats and θθθ *p *< 0.001 compared with diabetic rats.

Drug-induced diabetes is one of the most commonly used experimental diabetic models (28). In the present study diabetes was induced in rats by injection of streptozotocin. Diabetes is often accompanied by hyperlipidemia that manifests marked elevations of cholesterol, triglyceride, and LDL-C as well as low concentration of HDL-C (29, 30). These serum lipid abnormalities result due to disruption of fatty acid metabolism (31). Our results confirm that hyperlipidemia was occurred in the diabetic rats. Natural products that reduce or alter serum lipid profiles have proved to be effective for the treatment of many diabetic complications (32). Our findings showed that the oral administration of aqueous extract of *Cydonia oblonga* Mill. Fruit was able to ameliorate serum lipid profiles in the diabetic rats. It can be therefore suggested that quince fruit could be a potential source of hypolipidemic agent (s) and it can be used in the management of hyperlipidemia in diabetic patients.

Diabetes plays a central role in the initiation and progression of liver injury and this progressive disease is an independent risk factor for the development of chronic liver diseases (33, 34). The serum activities of ALT, AST, and ALP are biomarkers of hepatic injury (15, 35). ALT and AST are transaminase enzymes that catalyse amino transfer reactions and play an important role in amino acids catabolism and biosynthesis (36, 37). In addition, ALP is a hydrolase enzyme which acts as non-specific phosphomonoesterases to hydrolyse phosphate esters (38). In the present study, the serum elevation of liver damage biomarkers was occurred as a result of deleterious effect of hyperglycemia in the liver of diabetic rats. Increasing the activities of these enzymes is mainly due to leakage of the enzymes from the liver into the blood stream as a result of streptozotocin toxicity which leads to the liver damage. However, the treatment of diabetic groups with the extract of *Cydonia oblonga* Mill. for 42 consecutive days could ameliorate the activities of the above enzymes. A possible explanation for the hepatoprotective effects of the extract is that this fruit may inhibit the liver damage induced by streptozotocin. These results suggest a hepatoprotective role for quince fruit against liver injury associated with diabetes.

Diabetes mellitus is also associated with complications in the renal system. Patients with diabetes experience major long-term complications such as nephropathy and diabetic nephropathy is one of the leading causes of end-stage renal disease (ESRD) in the world (39, 40). Our results reconfirmed that the plasma levels of urea and creatinine, which are considered as significant biomarkers of renal dysfunction (41), were increased in the experimentally induced-diabetes. However, the treatment of diabetic rats with the extract of *Cydonia oblonga* Mill. reduced their plasma urea and creatinine levels. This implies that quince fruit normalizes the function of kidneys in the diabetic rats.

It was reported that the extract of *Cydonia oblonga* Mill. leaves possessed remarkable hypoglycemic effect in streptozotocin-induced diabetic rats. The leaves extract also showed antioxidant activity and protected the heart tissue against lipid peroxides produced by diabetes (23). In addition, *Cydonia oblonga* Mill. Leaf extracts showed hypolipidaemic and hepatoprotective effects in the rat model of hyperlipidaemia (22). Our results demonstrated that the fruit of* Cydonia oblonga* Mill. possesses hypolipidemic, hepatoprotective, and renoprotective effects in streptozotocin-induced diabetic rats. Previous studies have also shown that *Cydonia oblonga* Mill. Fruit contains polyphenols (19). It is well established that polyphenolic compounds have hypoglycemic activity and prevent the development of diabetic complications (42, 43). Therefore, the presence of these constituents may explain the protective effects of this fruit in diabetes-related complications. However, we believe that further studies are necessary to determine the exact nature of the active components and the mechanism of action of *Cydonia oblonga* Mill. Fruit in diabetes and its associated complications.

## Conclusion

The results of this study demonstrate that the oral administration of aqueous extract of *Cydonia oblonga* Mill. Fruit improve serum lipid profile in diabetic rats by lowering cholesterol, triglyceride, and LDL-C levels and raising HDL-C levels. In addition, the hepatoprotective effect of quince fruit is demonstrated by the significant reduction of serum levels of ALT, AST, and ALP in the diabetic treated rats. The extract also improved renal function in diabetic rats by reducing serum urea and creatinine. It can be concluded that *Cydonia oblonga* Mill. Fruit possesses hypolipidemic, hepatoprotective, and renoprotective effects in streptozotocin-induced diabetic rats.

## References

[1] Bilbis LS, Shehu RA, Abubakar MG (2002). Hypoglycemic and hypolipidemic effects of aqueous extract of Arachis hypogaea in normal and alloxan-induced diabetic rats. Phytomedicine.

[2] Irshaid F, Mansi K, Bani-Khaled A, Aburjia T (2012). Hepatoprotetive, cardioprotective and nephroprotective actions of essential oil extract of Artemisia sieberi in alloxan induced diabetic rats. Iran. J. Pharm. Res..

[3] Ashraf H, Heidari R, Nejati V, Ilkhanipoor M (2013). Effects of aqueous extract of Berberis integerrima root on some physiological parameters in streptozotocin-induced diabetic rats. Iran. J. Pharm. Res..

[4] Campos C (2012). Chronic hyperglycemia and glucose toxicity: pathology and clinical sequelae. Postgrad. Med..

[5] Lotfy M, Singh J, Hameed R, Tariq S, Zilahi E, Adeghate E (2013). Mechanism of the beneficial and protective effects of exenatide in diabetic rats. J. Endocrinol..

[6] Eliasson B, Gudbjörnsdottir S, Zethelius B, Eeg-Olofsson K, Cederholm J (2013). LDL-cholesterol versus non-HDL-to-HDL-cholesterol ratio and risk for coronary heart disease in type 2 diabetes. Eur. J. Prev. Cardiol..

[7] Byrne CD (2012). Non-alcoholic fatty liver disease, insulin resistance and ectopic fat: a new problem in diabetes management. Diabet. Med..

[8] El-Serag HB, Tran T, Everhart JE (2004). Diabetes increases the risk of chronic liver disease and hepatocellular carcinoma. Gastroenterology.

[9] Ikeda Y, Shimada M, Hasegawa H, Gion T, Kajiyama K, Shirabe K, Yanaga K, Takenaka K, Sugimachi K (1998). Prognosis of hepatocellular carcinoma with diabetes mellitus after hepatic resection. Hepatology.

[10] Inoue M, Tsugane S (2012). Insulin resistance and cancer: epidemiological evidence. Endocr- Relat. Cancer.

[11] Wang P, Kang D, Cao W, Wang Y, Liu Z (2012). Diabetes mellitus and risk of hepatocellular carcinoma: a systematic review and meta-analysis. Diabetes Metab. Res..

[12] Arkkila PE, Koskinen PJ, Kantola IM, Rönnemaa T, Seppänen E, Viikari JS (2001). Diabetic complications are associated with liver enzyme activities in people with type 1 diabetes. Diabetes Res. Clin. Pract..

[13] Antien L, Stephan JL, Diana C, Irene GM, Van V, Stefan B, Henk JG, Friedo W, Jan AB, Gerjan N, Bart J, Hans J (2010). Association between CNDP1 Ggenotype and diabetic nephropathy is sex specific. Diabetes.

[14] El-Demerdash FM, Yousef MI, El-Naga NI (2005). Biochemical study on the hypoglycemic effects of onion and garlic in alloxan-induced diabetic rats. Food Chem. Toxicol..

[15] Ahmadvand H, Tavafi M, Khalatbary AR (2012). Hepatoprotective and hypolipidemic effects of Satureja Khuzestanica essential oil in alloxan-induced type 1 diabetic rats. Iran. J. Pharm. Res..

[16] Odetola AA, Akinloye O, Egunjobi C, Adekunle WA, Ayoola AO (2006). Possible antidiabetic and antihyperlipidaemic effect of fermented Parkia biglobosa (JACQ) extract in alloxan-induced diabetic rats. Clin. Exp. Pharmacol. Physiol..

[17] World Health Organisation (1980). WHO Expert Committee on Diabetes Mellitus. Second Report. Technical Report Series 646.

[18] Yüksel C, Mutaf F, DemirtaåŸ I, Oztürk G, PektaåŸ M, Ergül A (2013). Characterization of Anatolian traditional quince cultivars, based on microsatellite markers. Genet. Mol. Res..

[19] Silva BM, Andrade PB, Martins RC, Valentão P, Ferreres F, Seabra RM, Ferreira MA (2005). Quince (Cydonia oblonga miller) fruit characterization using principal component analysis. J. Agric. Food Chem..

[20] Silva BM, Casal S, Andrade PB, Seabra RM, Oliveira MB, Ferreira MA (2004). Free amino acid composition of quince (Cydonia oblonga Miller) fruit (pulp and peel) and jam. J. Agric. Food Chem..

[21] Silva BM, Andrade PB, Mendes GC, Seabra RM, Ferreira MA (2002). Study of the organic acids composition of quince (Cydonia oblonga Miller) fruit and jam. J. Agric. Food Chem..

[22] Abliz A, Aji Q, Abdusalam E, Sun X, Abdurahman A, Zhou W, Moore N, Umar A (2013). Effect of Cydonia oblonga Mill. leaf extract on serum lipids and liver function in a rat model of hyperlipidaemia. J. Ethnopharmacol..

[23] Aslan M, Orhan N, Orhan DD, Ergun F (2010). Hypoglycemic activity and antioxidant potential of some medicinal plants traditionally used in Turkey for diabetes. J. Ethnopharmacol..

[24] Pourahmad J, Eskandari MR, Shakibaei R, Kamalinejad M (2010). A search for hepatoprotective activity of aqueous extract of Rhus coriaria L. against oxidative stress cytotoxicity. Food Chem. Toxicol..

[25] Juárez-Rojop IE, Díaz-Zagoya JC, Ble-Castillo JL, Miranda-Osorio PH, Castell-Rodríguez AE, Tovilla-Zárate CA, Rodríguez-Hernández A, Aguilar-Mariscal H, Ramón-Frías T, Bermúdez-Ocaña DY (2012). Hypoglycemic effect of Carica papaya leaves in streptozotocin-induced diabetic rats. BMC Complement. Altern. Med..

[26] Moradabadi L, Montasser Kouhsari S, Fehresti Sani M (2013). Hypoglycemic effects of three medicinal plants in experimental diabetes: inhibition of rat intestinal α-glucosidase and enhanced pancreatic insulin and cardiac Glut-4 mRNAs expression. Iran. J. Pharm. Res..

[27] Friedewald WT, Levy RI, Fredrickson DS (1972). Estimation of the concentration of low-density lipoprotein cholesterol in plasma without use of preparative-centrifuge. Clin. Chem..

[28] Sakata N, Yoshimatsu G, Tsuchiya H, Egawa S, Unno M (2012). Animal models of diabetes mellitus for islet transplantation. Exp . Diabetes Res..

[29] Howard BV (1987). Lipoprotein metabolism in diabetes mellitus. J. Lipid Res..

[30] Kim DK, Escalante DA, Garber AJ (1993). Prevention of atherosclerosis in diabetes: Emphasis on treatment for the abnormal lipoprotein metabolism of diabetes. Clin. Ther..

[31] Grover HS, Luthra S (2013). Molecular mechanisms involved in the bidirectional relationship between diabetes mellitus and periodontal disease. J. Indian Soc. Periodontol..

[32] Monday OM, Uzoma AI (2013). Histological changes and antidiabetic activities of Icacina trichantha tuber extract in beta-cells of alloxan induced diabetic rats. Asian Pac. J. Trop. Biomed..

[33] Hickman IJ, Macdonald GA (2007). Impact of diabetes on the severity of liver disease. Am. J. Med..

[34] Harrison SA (2006). Liver disease in patients with diabetes mellitus. J. Clin. Gastroenterol..

[35] Eidi A, Eidi M, Esmaeili E (2006). Antidiabetic effect of garlic (Allium sativum L.) in normal and streptozotocin-induced diabetic rats. Phytomedicine.

[36] Ayepola OR, Chegou NN, Brooks NL, Oguntibeju OO (2013). Kolaviron, a Garcinia biflavonoid complex ameliorates hyperglycemia-mediated hepatic injury in rats via suppression of inflammatory responses. BMC Complement. Altern. Med..

[37] Mossa AT, Refaie AA, Ramadan A, Bouajila J (2013). Amelioration of prallethrin-induced oxidative stress and hepatotoxicity in rat by the administration of Origanum majorana essential oil. Biomed. Res. Int..

[38] Kim EE, Wyckoff HW (1991). Reaction mechanism of alkaline phosphatase based on crystal structures. Two-metal ion catalysis. J. Mol. Biol..

[39] Kojima N, Slaughter TN, Paige A, Kato S, Roman RJ, Williams JM (2013). Comparison of the development diabetic induced renal disease in strains of Goto-Kakizaki rats. J. Diabetes Metab..

[40] Collins AJ, Foley RN, Herzog C, Chavers BM, Gilbertson D, Ishani A, Kasiske BL, Liu J, Mau LW, McBean M, Murray A, St Peter W, Guo H, Li Q, Li S, Li S, Peng Y, Qiu Y, Roberts T, Skeans M, Snyder J, Solid C, Wang C, Weinhandl E, Zaun D, Arko C, Chen SC, Dalleska F, Daniels F, Dunning S, Ebben J, Frazier E, Hanzlik C, Johnson R, Sheets D, Wang X, Forrest B, Constantini E, Everson S, Eggers PW, Agodoa L (2010). Excerpts from the US renal data system 2009 annual data report. Am. J. Kidney Dis..

[41] Ali BH, Al-Husseni I, Beegam S, Al-Shukaili A, Nemmar A, Schierling S, Queisser N, Schupp N (2013). Effect of gum arabic on oxidative stress and inflammation in adenine-induced chronic renal failure in rats. PLoS One.

[42] Ho CT, Wang M (2013). Dietary phenolics as reactive carbonyl scavengers: potential impact on human health and mechanism of action. J. Tradit. Complement. Med..

[43] Dong Q1, Banaich MS, O'Brien PJ (2010). Cytoprotection by almond skin extracts or catechins of hepatocyte cytotoxicity induced by hydroperoxide (oxidative stress model) versus glyoxal or methylglyoxal (carbonylation model). Chem. Biol. Interact..

